# From Free-Form to Framework: Evaluating the Effect of a Note Template on Emergency Medicine Residents’ Medical Decision-Making Documentation

**DOI:** 10.7759/cureus.100879

**Published:** 2026-01-05

**Authors:** Melissa Smith, Anthony Han, Ryan Ward, Philip Jarrett

**Affiliations:** 1 Emergency Medicine, UT Southwestern Medical Center, Dallas, USA

**Keywords:** billing and coding, clinical documentation, electronic health record, emergency medicine, medical decision-making, quality improvement, resident education

## Abstract

Introduction

Accurate and thorough medical decision-making (MDM) documentation is essential for quality patient care, inter-provider communication, and appropriate billing in emergency medicine (EM). However, resident notes often lack critical elements required for complete clinical and billing documentation. To address this gap, a structured MDM note template was developed and implemented at a large, urban academic ED to support EM residents in improving their documentation practices.

Methods

This retrospective quasi-experimental study evaluated senior resident charts from March to May 2022 (pre-intervention) and March to May 2024 (post-intervention). Following a one-year lead-in period with educational reinforcement, residents had the option to adopt the structured MDM template. Chart documentation was scored across 18 elements of MDM. A total of 641 charts met the inclusion criteria and were divided into three groups: pre-intervention (n = 257), post-intervention template users (TU; n = 195), and post-intervention non-template users (NTU; n = 189).

Results

Use of the structured template was associated with a significant increase in the median composite documentation score, rising from 5 (IQR 4-7) pre-intervention to 7 (IQR 5-8) among TU (p < 0.001). TU demonstrated significantly higher documentation rates in key categories compared to both pre-intervention and non-template groups, including interpretation of laboratory results (63.6% vs. 31.5% and 47.1%), ECG interpretation (29.7% vs. 16.7% and 16.4%), and consultant discussion (28.7% vs. 13.6% and 33.3%). NTU also showed improvement in some areas, likely reflecting the impact of concurrent educational efforts.

Conclusions

Implementation of a structured note template led to measurable improvements in EM residents’ MDM documentation. These gains were most pronounced in categories prompted by the template and suggest that combining documentation tools with targeted education may enhance documentation quality, optimize reimbursement, and support resident preparedness for independent clinical practice. Further research should explore long-term sustainability and integration with emerging technologies.

## Introduction

Accurate documentation is foundational to quality patient care, inter-provider communication, and appropriate reimbursement. However, notable differences exist between the documentation and billing practices of emergency medicine (EM) residents and attending physicians. Studies have shown that residents demonstrate lower billing accuracy than attendings, with one study reporting correct billing codes in only 37.5% of resident charts compared to 55.3% for attendings [1]. Residents are also more likely to miss billable elements and undercode patient encounters, resulting in significant revenue loss and failing to reflect the true complexity of care provided [2,3].

A key contributor to this discrepancy is variation in documentation completeness. Residents often fail to document faculty involvement or omit critical elements of medical decision-making (MDM), leading to potential compliance concerns and downcoding [4]. In contrast, attending physicians tend to produce more thorough documentation, which is associated with more accurate billing and helps prevent revenue loss due to downcoding [2].

Incomplete documentation not only affects reimbursement but also has clinical implications. Insufficient charting may obscure the complexity of patient presentations, underrepresent provider workload, and potentially impact downstream care decisions or quality metrics [5]. Studies have shown that improved documentation is associated with higher assigned evaluation and management (E/M) levels and greater reimbursement, underscoring the importance of documentation education in residency training [6].

Despite this, many EM residents report feeling underprepared in billing and documentation. Surveys indicate that fewer than 5% of residents feel extremely confident in their billing abilities, and over 80% are unsure about the cost of services rendered in the ED [7]. Faculty perceptions mirror these findings, with the majority agreeing that residents are inadequately trained in this domain [8].

Educational interventions have shown promise in addressing this gap. Strategies such as interactive lectures, team-based learning, and biweekly feedback have been associated with measurable improvements in documentation quality and relative value units generated per hour [6,9]. However, barriers remain. Many residency programs lack formal curricula on billing and coding, and the time constraints and acuity of the ED can limit opportunities for structured feedback [10].

Compounding these challenges, in 2023, the Centers for Medicare & Medicaid Services (CMS) introduced significant changes to ED E/M coding guidelines. These updates shifted the focus away from history and physical examination elements, emphasizing the complexity of MDM instead as the primary determinant of visit level [11]. These changes raise the stakes for documentation accuracy and heighten the need for resident education and tools that support high-quality MDM documentation.

To date, limited research has examined documentation tools specifically designed for residents in response to recent CMS changes. Although other studies have evaluated template-based documentation across inpatient, outpatient, and ED settings, they have focused on metrics such as note length and time spent charting [12,13]. There is little evidence on whether targeted note templates can improve documentation quality among EM residents. In this study, we introduced a structured note template intended to support high-quality MDM documentation. Our aim was to assess whether the template led to improvements in EM resident MDM documentation over a three-year retrospective period.

## Materials and methods

Setting

This study was conducted at a large, urban county hospital that serves as a primary teaching site for an academic medical institution. The study focused on patient encounters in the hospital’s ED, which manages approximately 220,000 visits annually. The affiliated academic institution hosts a three-year EM residency program with 26 residents per class.

Study design and data collection

This was a retrospective quasi-experimental study designed to evaluate whether implementation of a structured note template improved the quality of resident MDM documentation. The study was approved by an institutionally recognized quality improvement committee to enhance resident documentation quality; therefore, IRB approval was not sought or required. The study was conducted and reported in accordance with the Standards for Quality Improvement Reporting Excellence (SQuIRE 2.0) guidelines.

The project period spanned from March 2022 through May 2024. Prior to the introduction of the note template, resident charts were retrospectively audited from March to May 2022. Charts were selected sequentially by date, with sampling standardized by weekday, shift time, and ED treatment area to minimize variation in operational flow. Only senior resident charts were included, defined as residents in the last three months of their second or third postgraduate year (PGY2 and PGY3). The proportion of PGY2 and PGY3 resident authorship was similar across cohorts.

Resident reviewers, who were senior EM residents in their second or third year of training, underwent structured instruction on a predefined scoring rubric used to assess the quality and completeness of MDM documentation. Training included standardized grading rules and instructions for evaluating each chart. Each MDM element was scored as a binary outcome (present or absent), with reviewers directed to seek clarification as needed. Reviewers evaluated charts for the presence of specific MDM elements, awarding one point for discussion of comorbidities, review of prior chart information, inclusion of a differential diagnosis, listing of any labs ordered, interpretation of multiple laboratory results, listing of imaging ordered, interpretation of imaging results, medications ordered, discussion of high-risk medications administered, past medical and surgical histories, documentation of consultant involvement discussions, ECG ordering, ECG interpretation, critical care time, documentation of patient reassessment(s), and final disposition. Each chart was then assigned a composite MDM score based on the number of included elements.

In February 2023, the electronic medical record system’s resident note interface was updated to include default structural elements intended to enhance MDM documentation, in alignment with recent CMS guideline changes. Specific prompts, subcategories, and free-text boxes within the template were organized to address four main areas. The first area focused on chart review complexity, including documentation of an independent historian, external data review, and ordering and interpretation of laboratory results, radiology studies, and ECGs. The second area focused on risk management, including drug management and treatment disposition. The third area included free-text fields for an MDM narrative and discussion of management or test interpretation with external providers, such as consultants. The fourth area captured critical care documentation, including fields for time entry and descriptive elements.

Residents had the option to use the new default MDM template or continue using their personalized templates. No chart data were collected during 2023 to allow a lead-in period for adaptation. During this time, resident education on MDM documentation was reinforced through two dedicated lectures at the weekly didactic conference, focusing on recent CMS changes. Additionally, quarterly lectures reviewed anonymized resident charts and highlighted opportunities for improved billing and coding documentation.

From March to May 2024, a second round of chart reviews was conducted using the same weekday, shift time, and treatment area sampling strategy employed in 2022. Charts were categorized based on whether residents used the updated note template. The same scoring system for MDM elements was applied to assess the combined impact of template implementation and ongoing educational efforts.

Inclusion and exclusion criteria

Charts were included if they were authored by a senior EM resident, the encounter occurred in the main adult ED, and the encounter took place during a predefined weekday, shift time, and treatment area. All attending physician documentation, including any MDM documented in the attending attestation, was excluded. Patient encounters that began in treatment areas located on-site but outside the main adult ED, such as psychiatry, urgent care, or emergency obstetrics and gynecology, were also excluded. All patient encounters were unique, and any duplicate medical record numbers or charts were removed.

Outcome measures

A summed composite quality score was calculated for each encounter by adding the binary indicators from each of the predefined MDM elements, with a score of 1 for “yes” and 0 for “no.” Scores ranged from 0 to 13, with higher values indicating more complete documentation. Score distributions in the pre-implementation, post-implementation without-template, and post-implementation with-template groups were summarized using Tukey notched box plots, displaying medians, IQRs, 1.5 × IQR whiskers, and individual outliers. Pairwise differences in summed scores were evaluated with two-sided Mann-Whitney U tests, with nonoverlapping notches and p < 0.05 denoting statistical significance.

To explore potential reviewer effects, summed scores were stratified by reviewer. Group distributions were displayed with notched box plots ordered by median score, and a Kruskal-Wallis H test compared rank distributions across all reviewers. A significant H statistic (p < 0.05) would suggest that at least one reviewer’s scoring pattern differed from the others. Because each note was reviewed by only one reviewer, inter-rater reliability coefficients could not be calculated.

## Results

A total of 681 charts were screened, and 40 were excluded based on predetermined criteria, leaving 641 charts for final analysis. The pre-intervention group (March to May 2022) comprised 257 charts, while the post-intervention group (March to May 2024) included 384 charts, subdivided into 195 charts from template users (TU) and 189 charts from non-template users (NTU). Documentation was assessed across 18 discrete categories, each scored as either present (1) or absent (0) for each chart. One category, critical care time, was excluded from inferential testing in the primary endpoint due to zero documentation across all groups.

**Figure 1:**
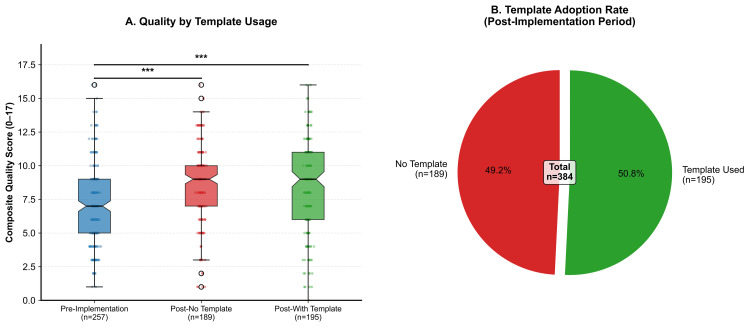
Template usage and quality scores (A) Tukey box plot comparing unweighted composite quality scores among pre-implementation, post-implementation without-template, and post-implementation with-template encounters. Medians, IQRs, sample sizes, and significant differences are shown. Group-wise comparisons were calculated using the Mann-Whitney U test. (B) Template adoption during the post-implementation period, showing counts and percentages of encounters documented with versus without the study template.

For the primary endpoint, summed composite quality scores increased markedly after the intervention. Pre-implementation encounters had a median score of 7 points (IQR 5-9), whereas post-implementation encounters reached a median of 9 points (IQR 7-11). The Hodges-Lehmann estimate of location shift was 2.0 points (95% CI: 1.0-3.0). This difference was statistically significant (Mann-Whitney U, p < 0.001) with a moderate effect size (Cohen’s d = 0.49, 95% CI: 0.33-0.65). Figure 1 illustrates these distributions alongside the post-implementation adoption rate of the structured MDM template.

Table 1 presents documentation frequencies and comparative statistics across groups, including chi-square test statistics (χ²) and p-values. Compared to the pre-intervention group, TU demonstrated statistically significant improvements in documentation for several categories prompted by the new template. These included interpretation of any lab result (63.6% vs. 31.5%, χ² = 44.73, p < 0.001), interpretation of multiple labs (47.2% vs. 23.7%, χ² = 26.18, p < 0.001), independent ECG interpretation (29.7% vs. 16.7%, χ² = 10.08, p = 0.001), and discussion with a consultant (28.7% vs. 13.6%, χ² = 14.80, p < 0.001). Notably, categories with embedded prompts in the template were among those with the most substantial gains.

**Table 1:** Documentation frequency and statistical comparison across groups This table displays documentation frequencies across key clinical categories among resident physicians in three groups: pre-intervention (Pre), template users (TU), and non-template users (NTU). The first three columns show raw counts and percentages. The final three columns present chi-square test results (χ²) comparing the following group pairs: TU vs. Pre, NTU vs. Pre, and TU vs. NTU. p-Values indicate the probability that observed differences occurred by chance, with p < 0.05 considered statistically significant. Categories marked with double asterisks (^**^) indicate elements prompted by structured fields in the documentation template. A dash (-) denotes comparisons not performed due to zero counts in all groups. PMH, past medical history; PSH, past surgical history

Category	Pre (n = 257)	TU (n = 195)	NTU (n = 189)	TU vs. Pre (χ², p)	NTU vs. Pre (χ², p)	TU vs. NTU (χ², p)
Discussion of comorbidities	162 (63.0%)	128 (65.6%)	147 (77.8%)	χ² = 0.22, p = 0.636	χ² = 10.44, p = 0.001	χ² = 6.37, p = 0.012
Documentation of chart review^**^	102 (39.7%)	64 (32.8%)	120 (63.5%)	χ² = 1.96, p = 0.161	χ² = 23.74, p < 0.001	χ² = 34.96, p < 0.001
Documented differential diagnosis list	173 (67.3%)	131 (67.2%)	156 (82.5%)	χ² = 0.00, p = 1.000	χ² = 12.27, p < 0.001	χ² = 11.19, p < 0.001
Listed multiple labs (three or more)^**^	149 (58.0%)	113 (57.9%)	125 (66.1%)	χ² = 0.00, p = 1.000	χ² = 2.73, p = 0.099	χ² = 2.39, p = 0.122
Interpreted any lab result^**^	81 (31.5%)	124 (63.6%)	89 (47.1%)	χ² = 44.73, p < 0.001	χ² = 10.55, p = 0.001	χ² = 9.92, p = 0.002
Interpreted multiple labs (two or more)^**^	61 (23.7%)	92 (47.2%)	67 (35.4%)	χ² = 26.18, p < 0.001	χ² = 6.74, p = 0.009	χ² = 4.97, p = 0.026
Documented imaging studies obtained^**^	153 (59.5%)	120 (61.5%)	126 (66.7%)	χ² = 0.11, p = 0.738	χ² = 2.07, p = 0.150	χ² = 0.88, p = 0.347
Documented note on the author’s independent imaging interpretation^**^	45 (17.5%)	49 (25.1%)	26 (13.8%)	χ² = 3.46, p = 0.063	χ² = 0.88, p = 0.347	χ² = 7.19, p = 0.007
Documented obtaining an ECG^**^	83 (32.3%)	82 (42.1%)	66 (34.9%)	χ² = 4.14, p = 0.042	χ² = 0.23, p = 0.632	χ² = 1.77, p = 0.183
Documented independent ECG interpretation^**^	43 (16.7%)	58 (29.7%)	31 (16.4%)	χ² = 10.08, p = 0.001	χ² = 0.00, p = 1.000	χ² = 8.86, p = 0.003
Documented placing a consult^**^	91 (35.4%)	84 (43.1%)	87 (46.0%)	χ² = 2.43, p = 0.119	χ² = 4.69, p = 0.030	χ² = 0.23, p = 0.631
Documented discussion with consultant^**^	35 (13.6%)	56 (28.7%)	63 (33.3%)	χ² = 14.80, p < 0.001	χ² = 23.55, p < 0.001	χ² = 0.75, p = 0.386
Discussed high-risk medications	15 (5.8%)	41 (21.0%)	39 (20.6%)	χ² = 22.19, p < 0.001	χ² = 21.04, p < 0.001	χ² = 0.00, p = 1.000
Discussed reassessment	52 (20.2%)	74 (37.9%)	69 (36.5%)	χ² = 16.44, p < 0.001	χ² = 13.78, p < 0.001	χ² = 0.03, p = 0.852
Discussed disposition^**^	127 (49.4%)	125 (64.1%)	121 (64.0%)	χ² = 9.11, p = 0.003	χ² = 8.83, p = 0.003	χ² = 0.00, p = 1.000
Critical care time documentation^**^	0 (0.0%)	0 (0.0%)	0 (0.0%)	-	-	-
Documentation of PMH in the PMH section of the note	225 (87.5%)	161 (82.6%)	148 (78.3%)	χ² = 1.83, p = 0.176	χ² = 6.14, p = 0.013	χ² = 0.85, p = 0.356
Documentation of PSH in the PSH section of the note	197 (76.7%)	144 (73.8%)	126 (66.7%)	χ² = 0.33, p = 0.564	χ² = 4.95, p = 0.026	χ² = 2.04, p = 0.153

Documentation among NTU also improved in several categories compared to the pre-intervention group, such as chart review (63.5% vs. 39.7%, χ² = 23.74, p < 0.001) and discussion with a consultant (33.3% vs. 13.6%, χ² = 23.55, p < 0.001), although in many cases these improvements were not statistically superior to those observed in the TU group. Direct comparison between post-intervention TU and NTU revealed differences favoring template use in key areas, including independent ECG interpretation (29.7% vs. 16.4%, χ² = 8.86, p = 0.003), independent imaging interpretation (25.1% vs. 13.8%, χ² = 7.19, p = 0.007), and interpretation of lab results (63.6% vs. 47.1%, χ² = 9.92, p = 0.002). These findings suggest that use of the standardized note template was associated with enhanced documentation, particularly in categories explicitly prompted by the template.

**Figure 2:**
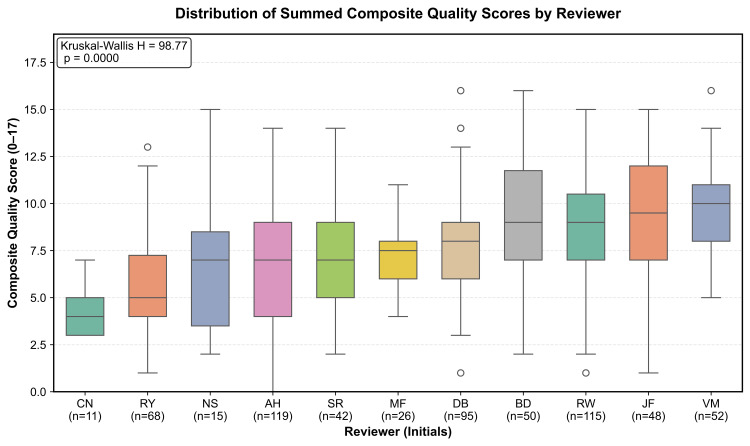
Reviewer-level distribution of summed quality scores Box plots illustrate composite-score distributions assigned by each reviewer, showing medians, IQRs, whiskers (1.5 × IQR), outliers, and per-reviewer sample sizes. A Kruskal-Wallis test (H statistic and p-value inset) evaluates whether score distributions differ across reviewers.

Summed composite scores differed appreciably across reviewers (Figure 2). Medians ranged from 4 to 10 points, and several reviewers displayed wider IQRs and higher upper whiskers, suggesting more generous scoring. A Kruskal-Wallis test confirmed that at least one reviewer’s distribution diverged significantly from the others (H = 98.77, p < 0.001), indicating systematic variability in the application of quality criteria. Although encounter complexity was not independently measured, these differences highlight potential reviewer bias and underscore the need to adjust for or explicitly discuss reviewer effects when interpreting observed improvements in documentation quality.

## Discussion

The findings of this study indicate that implementation of a structured MDM note template was associated with measurable improvements in documentation quality among EM residents. Residents who utilized the template achieved higher composite documentation scores and demonstrated increased documentation rates across several key MDM categories, particularly those directly prompted by the template. These results are consistent with existing literature on the benefits of structured documentation tools, which have been shown to enhance note completeness and standardization in both inpatient and outpatient care settings [14,15].

Notably, improvements were not limited to residents who used the template. NTU in the post-intervention cohort also demonstrated increased documentation rates in multiple categories, suggesting that concurrent educational efforts contributed meaningfully to overall documentation quality [16]. Nevertheless, the magnitude of improvement observed among TU, especially in areas such as laboratory interpretation, ECG interpretation, and consultant communication, supports the utility of embedded prompts in closing documentation gaps not fully addressed by education alone.

Structured templates may also serve an educational function beyond compliance with billing standards. As residents develop clinical reasoning and documentation skills, structured prompts can reinforce best practices and encourage more complete representations of diagnostic complexity and care decisions [7,17,18]. In this way, templates may act as cognitive scaffolds, supporting both real-time charting and longitudinal skill development.

Despite these gains, certain areas, such as documentation of critical care time and high-risk medication use, remained underrepresented. These omissions suggest that template design and educational interventions may benefit from additional refinement to address documentation areas that are both clinically and financially significant. Incorporating regular chart audits and individualized feedback may help reinforce adherence and address persistent deficiencies [19].

Several limitations should be considered. First, because the pre- and post-intervention groups were separated by two academic years, this study reflects a historical control rather than a true within-subject comparison. Although PGY2/PGY3 representation was similar and charts were sampled using standardized weekdays, shift times, and treatment areas, encounter complexity and admission rates were not tracked, which may introduce unmeasured confounding. Two of the chart elements analyzed, past medical history and past surgical history, are no longer part of the revised CMS E/M billing framework and may not reflect documentation behaviors relevant to current billing practices. Additionally, collateral history obtained from independent historians, which contributes to visit complexity, was not captured in this study.

Use of the template was voluntary, introducing the possibility of self-selection bias. Residents who chose not to use the template may differ from users in motivation, organization, or engagement with documentation expectations, which could contribute to higher or lower scores independent of the intervention itself. The concurrent timing of template implementation and educational sessions limits the ability to isolate the effect of either intervention alone. Finally, observed variability in scoring across reviewers may have introduced unmeasured bias and warrants further exploration in future studies.

Taken together, these findings support the use of structured documentation tools in residency training as a strategy to enhance documentation quality and compliance. The template appeared to address common gaps in resident documentation and, when paired with dedicated instruction, may facilitate more accurate and complete MDM charting [20,21].

This carries particular relevance as EDs face increased pressure to demonstrate value and recover appropriate reimbursement. The anticipated surplus in the EM workforce, along with evolving payment models, underscores the importance of documentation practices that accurately reflect patient complexity and provider decision-making [22,23]. Structured documentation tools may also help prepare residents for the future integration of AI in clinical documentation. As AI-driven tools begin to auto-populate or suggest billing-relevant content, the role of the resident may evolve from note author to content editor and verifier [24-27]. In this context, templates provide an important transitional tool, enhancing accuracy now while cultivating the documentation literacy needed to engage effectively with AI systems.

Ultimately, documentation training should be regarded as a foundational component of EM education. The combined use of structured templates, targeted teaching, and evolving technology offers a practical approach to equipping residents with the skills necessary for accurate, compliant, and clinically reflective documentation.

## Conclusions

The introduction of a structured MDM note template led to measurable improvements in EM resident documentation. Residents using the template demonstrated enhanced chart completeness in critical areas of MDM, while educational sessions contributed to improvements even among NTU. These findings highlight how structured documentation tools, combined with targeted teaching, can work synergistically to support resident development and improve the quality of clinical notes.

As documentation standards and technologies continue to evolve, residency programs must remain adaptable to prepare trainees for the demands of modern clinical practice. Embedding educational frameworks within clinical tools not only enhances real-time learning but also positions programs to integrate emerging innovations, such as AI-assisted documentation, in the future. Thoughtful incorporation of such technologies may further support accuracy and efficiency without replacing the essential clinical reasoning skills residents need to develop. Ultimately, investing in comprehensive documentation education is critical for fostering competent, future-ready physicians equipped to navigate an increasingly complex healthcare landscape.
